# SARS-CoV-2 Neutralizing Antibodies in White-Tailed Deer from Texas

**DOI:** 10.1089/vbz.2021.0094

**Published:** 2022-01-13

**Authors:** Pedro M. Palermo, Jeanette Orbegozo, Douglas M. Watts, John C. Morrill

**Affiliations:** ^1^ Department of Biological Sciences, Border Biomedical Research Center, University of Texas at El Paso, El Paso, Texas, USA.; ^2^ Orion Research and Management Services, Gatesville, Texas, USA.

**Keywords:** SARS-CoV-2, antibodies, white-tailed deer, Texas

## Abstract

Serological evidence of SARS-CoV-2 infection among white-tailed deer has been reported from Illinois, Michigan, Pennsylvania, and New York. This study was conducted to determine whether deer in Texas also had evidence of SARS-CoV-2 infection. Archived sera samples collected from deer in Travis County, Texas, during 2018, before and during the pandemic in 2021 were tested for neutralizing antibody to this virus by a standard plaque reduction neutralization assay. SARS-CoV-2 antibody was not detected in 40 deer sera samples collected during 2018, but 37% (20/54) samples collected in 2021 were positive for antibody. The seroprevalence rate between males and females differed significantly (*p* < 0.05) and the highest rate (82%) was detected in the 1.5-year-old animals. These findings extended the geographical range of prior SARS-CoV-2 infection among white-tailed deer in the United States and further confirm that infection was common among this species.

## Introduction

Evidence of natural SARS-CoV-2 infections has been reported in dogs, cats, mink, tigers, and lions in Hong Kong, Netherlands, China, and the United States, and more recently in white-tailed deer in the United States (McAloose et al. 2020, Oreshkova et al. 2020, Chandler et al. 2021, Sit et al. 2020). Also, white-tailed deer were susceptible to experimental infection with SARS-CoV-2 and the virus was transmitted to uninfected deer that were caged with the infected animals (Palmer et al. 2021).

Even though these domestic and wild animals were susceptible to SARS-CoV-2 infection and some develop symptomatic infections, none have been implicated as a source of human infection. To further understand the geographical distribution of SAS-CoV-2 infected deer, this study was conducted to determine whether there was serological evidence of infection by this virus among deer in Travis County, Texas.

## Materials and Methods

### Sample collection

A total of 54 blood samples were collected from hunter-harvested deer from January to February, 2021, as part of a zoonotic disease surveillance study in several areas of Travis County, Texas. The samples were collected in accordance with Scientific Permit SPR-081 issued by the Texas Park and Wildlife Department, Austin, Texas. Another subset of 40 deer blood samples from Travis County in 2018 was also considered in the study (Palermo et al. 2020). The date and location of collection, and gender and age were recorded for each deer. The age of deer was determined by dental examination (Cain and Wallace 2003). Blood samples were obtained by postmortem cardiac puncture at a central collecting station and transported to the laboratory for processing. Samples were centrifuged at 1200 × *g* at 4°C for 10–15 min and then the sera were stored in aliquots of 1 to 2 mL at 20°C until tested for SARS-CoV-2 neutralizing antibody.

### SARS-CoV-2 neutralization test

Deer sera were tested for neutralizing antibody to SARS-CoV-2. In brief, after 1:5 dilution, the heat-inactivated sera samples were incubated at 37°C for 1 h with 40–50 plaque-forming units (PFUs) of SARS-CoV-2 (USA-WA1/2020 strain) suspensions. Sera samples and virus were diluted in Eagle's minimum essential's medium containing 2% fetal bovine serum and 1% penicillin/streptomycin. After incubation, 100 μL of the sera and virus mixtures was inoculated in duplicates onto monolayers of Vero E6 cells grown in 12-well plates and incubated for 1 h at 37°C and 5% CO_2_.

Then, 0.8% agarose was added onto the cell monolayer and the cells were incubated for 2 days at 37°C and 5% CO_2_ and then stained with a 0.03% neutral red solution. The virus dose was determined as the mean number of PFUs recorded on 12-well cells infected with 40–50 PFUs based on the testing of the viral dose dilution and negative control. The PFUs were counted and if the sera dilution (1:10) reduced ≥80% of the virus dose, the sample was considered positive for antibody. A chi-square test was used to compare antibody seroprevalence rates among gender and age group. All the statistical analyses were performed using GraphPad 8.0 (San Diego, CA).

## Results

Among the 54 deer sera samples collected from January to February 2021 or during the COVID-19 pandemic, 37% (*n* = 20) were positive for SARS-CoV-2 neutralizing antibody. In contrast, SARS-CoV-2 neutralizing antibody was not detected in the 40 deer sera samples collected during 2018 or during the prepandemic period. A significant difference (*p* < 0.05) in the SARS-CoV-2 seroprevalence rates was observed between males (15/30, 50%) and females (5/24, 21%).

**FIG. 1.**
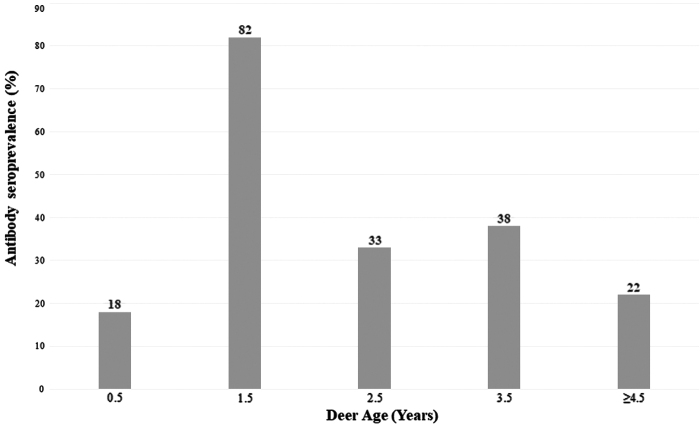
Prevalence of SARS-CoV-2 antibody in white-tailed deer by age in Travis County during the SARS-CoV-2 pandemic.

An average of 10.8 deer sera samples were tested in each age group, including 0.5, 1.5, 2.5, 3.5, and >4.5 years, with more sera samples (*n* = 17) tested for the 0.5 year group. The highest SARS-CoV-2 seroprevalence rate was 82% (9/11) among the 1.5 years age group (Fig. 1).

## Discussion and Conclusions

Our study is the first to report serological evidence of SARS-CoV-2 infection in deer from Texas and extended the previously reported area of SARS-CoV-2 antibody among this animal in the United States (Chandler et al. 2021). The 37% antibody seroprevalence observed in this study was comparable with the 40% rate reported in the four mid-western states and northeast United States. Also, SARS-CoV-2 neutralizing antibody was detected in experimentally infected deer (<0.5 to 2 years) on day 7 postinfection (dpi), and the antibody titers increased from 14 dpi to 21 dpi (Cool et al. 2021, Palmer et al. 2021).

SARS-CoV-2 neutralizing antibody was not detected in deer sampled in Travis County during 2018 or before the SARS-CoV-2 pandemic, indicating that the deer were not infected by SARS-CoV-2 or other coronavirus(es) that could have caused serological cross-reactivity during this period. Similar negative results were also reported for deer sera collected before 2020 in the United States (Chandler et al. 2021).

The SARS-CoV-2 seroprevalence rate varies by age and was higher (82%) in deer 1.5-year-old, but was based on a limited number of tested samples in each age group. Deer live in small herds, such as females and fawns' herd together and males form small herds. This social behavior in deer may increase their direct or indirect contact, which has been demonstrated to be efficient for SARS-CoV-2 transmission during a 48-h period (Cool et al. 2021, Palmer et al. 2021) and could explain the variation of the SARS-CoV-2 seroprevalence rate by age. Although the source of SARS-CoV-2 infection among deer is not understood, the findings of this study extended the geographical range of prior SARS-CoV-2 infected deer to Texas.

Recent studies have suggested humans as source of SARS-CoV-2 infection of deer (Kuchipudi et al. 2021). However, the possible mechanism(s) of transmission from humans to deer remain unknown. Also, the epidemiological significance of infection of deer is yet to be determined but warrants consideration as a possible reservoir in view of the hypothesis that SARS-CoV-2 infection of humans originated from infected animals (Von Borowski and Trentin 2021).

Therefore, as one of the most abundant wildlife species, and the rapidly growing population and invasion of rural and urban communities, deer are not to be neglected as a possible source of SARS-CoV-2 infection of human and domestic and wildlife animals. An understanding of the environmental and ecological factors that serve as determinants of the acquisition of SARS-CoV-2 infection and spread among deer is needed to mitigate any risk(s) associated with deer as a possible reservoir of human infection.
